# A meta‐analysis on the heritability of vertebrate telomere length

**DOI:** 10.1111/jeb.14071

**Published:** 2022-08-06

**Authors:** Heung Ying Janet Chik, Alexandra M. Sparks, Julia Schroeder, Hannah L. Dugdale

**Affiliations:** ^1^ Groningen Institute for Evolutionary Life Sciences University of Groningen Groningen The Netherlands; ^2^ School of Natural Sciences Macquarie University Sydney New South Wales Australia; ^3^ Faculty of Biological Sciences, School of Biology University of Leeds Leeds UK; ^4^ School of Biosciences University of Sheffield Sheffield UK; ^5^ Department of Life Sciences Imperial College London Silwood Park Ascot UK

**Keywords:** heritability, meta‐analysis, quantitative genetics, senescence, systematic review, telomere length

## Abstract

Telomere dynamics are linked with both cellular and organismal senescence, and life history, individual quality and health. Telomere dynamics, particularly telomere length, have therefore garnered much research interest in evolutionary biology. To examine the evolution of telomere length, it is important to quantify its heritability, the proportion of total variation explained by additive genetic effects. Many studies have quantified telomere length heritability, but estimates are varied, and no general conclusion has been drawn. Additionally, it is unclear whether biological and methodological factors influence telomere length heritability estimates. We present the first meta‐analysis of telomere length heritability, using 104 estimates from 43 studies over 18 vertebrate species. We calculated an overall mean heritability and examined how estimates varied by study, phylogeny, species‐specific ecology, environmental setting, age at sampling, laboratory methods, statistical methods, sex and repeated measurements. Overall heritability was moderate (44.9%, 95% CI: 25.2–64.7%), and there was considerable heterogeneity in heritability estimates, in particular among studies and estimates. Laboratory method influenced heritability estimates, with in‐gel hybridization TRF yielding higher heritabilities than qPCR and Southern blot TRF. There was also an effect from statistical method, with twin‐based and SNP‐based estimates lower than correlation‐based or pedigree‐based estimates. Our results highlight an overall heritable basis of telomere length, and we recommend future research on a wider range of taxa, and the use of variance‐partitioning methods with relatedness or SNP data over correlation methods to minimize heritability estimation bias.

## Graphical abstract

In this meta‐analysis, we found that telomere length has a moderate heritability of 44.9%, and heritability estimates are influenced by laboratory and statistical methods.

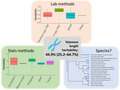

## 1 INTRODUCTION

Telomeres are non‐coding, repeating DNA sequences that cap the ends of chromosomes and serve important functions, including maintaining chromosome stability and protecting chromosomal DNA from erosion (Blackburn & Szostak, 1984; Campisi, 2005). Telomeres shorten over time due to the end replication problem, where the ends of DNA strands are not fully replicated during cell division (Levy et al., 1992), and also due to stressors, such as oxidative stress or UV light (Monaghan & Ozanne, 2018). Such shortening can be rescued by, among other mechanisms, the enzyme telomerase, which adds telomeric repeats to chromosome ends to counterbalance telomere loss, but telomerase is generally suppressed in adult cells (reviewed in Haussmann & Treidel, 2015). When telomeres reach a critical length, the cell either enters replicative arrest or undergoes cell death, linking telomere length with cellular senescence and other physiological deteriorations (Campisi, 2005; Monaghan et al., 2008). Short telomeres are also linked with increased mortality (Wilbourn et al., 2018), decreased lifespan (Bichet et al., 2020; Eastwood et al., 2019; Heidinger et al., 2012), decreased reproductive success (Bauch et al., 2020; Eastwood et al., 2019; Parolini et al., 2017) and increased age‐related disease risk (Aviv & Shay, 2018; Cawthon et al., 2003; Zee et al., 2010). As such, telomere dynamics have been frequently used as biomarkers of senescence, individual quality and health, in evolutionary biology.

To understand the evolutionary response to selection on telomere length, it is crucial to know whether telomere length is heritable. Although telomeres are directly inherited by offspring from their parents, telomere length at any one time is also influenced by the rate and amount of shortening, as well as of elongation, both of which could be influenced by multiple genes, and/or the environment (Dugdale & Richardson, 2018). Instead of identifying the genetic variants causally linked to telomere length, quantitative genetics offers a useful analytical framework to summarize the contribution of (additive) genetic effects to the observed phenotypic variation, relative to the contribution of environmental effects. This is done via the heritability metric, defined as the proportion of additive genetic variance to total phenotypic variance (Falconer & Mackay, 1996). A non‐zero heritability would indicate the presence of a genetic basis of telomere length, and thus the potential to evolve under selection (Lynch & Walsh, 1998). Given the interest in telomere biology, the heritability of telomere length has been estimated in a wide range of taxa (reviewed in Atema et al., 2015; Bauch et al., 2021; Dugdale & Richardson, 2018). However, these heritability estimates ranged from 0% to 100%, and hence, no general conclusions regarding the evolutionary potential of telomere length could be drawn (Dugdale & Richardson, 2018).

Such large variation among telomere length heritability estimates is likely to reflect true biological variation between populations. First, as telomere length is coupled with fitness (Eastwood et al., 2019), it is expected that telomere length is under different selection pressures depending on the environment, which would influence genetic variance, and consequently heritability, among species to different degrees (Mousseau & Roff, 1987; Walsh & Lynch, 2018). This would manifest as phylogenetic non‐independence, where closely related, more recently diverged species would have more similar heritability estimates than distant species. Second, as heritability measures the relative contribution of genes versus the environment, one can expect it to be space‐, time‐ and context‐dependent. For example, provided that all individuals are sampled at the same age, heritability can be expected to be higher in a stable environment (e.g. in a laboratory), than in a varied environment (e.g. in the wild), because in the former environmental variation is minimized, and observed among‐individual telomere length differences must then come from genetic differences, resulting in heritability being closer to 100% (Dugdale & Richardson, 2018). In addition, the duration of environmental exposure increases as individuals age. As telomere length is altered by extrinsic stressors (Monaghan & Ozanne, 2018), the relative contribution of the environment to telomere length variation would increase with age, even if the environment remains constant, leading to a predicted decrease in heritability estimates with sampling age (Dugdale & Richardson, 2018).

Perhaps more concerning is that heritability estimates can also be influenced by methodological factors. For example, different telomere measurement methods yield different technical telomere length repeatabilities. For example, measuring terminal restriction fragments (TRF) yield more repeatable telomere lengths than quantitative PCR (qPCR; Aviv et al., 2011; Montpetit et al., 2014) due to the amplification of errors (Nettle et al., 2019) and the inclusion of interstitial telomeric sequences (ITS) in the latter (Foote et al., 2013). Within TRF methods, in‐gel hybridization does not include ITS, but Southern blotting does (Foote et al., 2013). A recent meta‐analysis put the repeatability of TRF at 0.80 (95% CI = 0.34–0.96) compared with 0.46 (95% CI = 0.04–0.82) for qPCR (Karkkainen et al., 2021). Given TRF generally has higher technical repeatability, we would expect heritability estimates from TRF studies to be higher than those from qPCR studies. Perhaps more importantly, selection of statistical methods could influence heritability estimates. In many studies, heritability is measured using parent–offspring regression, where offspring trait values are regressed against the parental trait values (either of the mother, the father, or the average of both, termed ‘mid‐parent’), and the slope indicates parent–offspring trait similarity and thus heritability (Falconer & Mackay, 1996). Alternatively, heritability can be derived from phenotypic correlations between siblings as well in a similar manner (Falconer & Mackay, 1996). Both methods, however, do not account for any similarity arising from shared environments, which could be inseparable from genetic effects (Kruuk & Hadfield, 2007) and lead to overestimation of heritability. Quantitative genetic ‘animal’ models aim to offer a solution to these caveats, by using genetic or social pedigrees to partition phenotypic variance into genetic and various environmental components (Wilson et al., 2010). However, to separate additive genetic variance and permanent environmental variance, that is variance arising from the unique circumstances of each individual, multiple measurements per individual are required, without which heritability estimates would be inflated (Wilson et al., 2010). In addition, the accuracy of heritability estimates from pedigree‐based methods would also depend on pedigree structure, accuracy and completeness, in addition to the explicit inclusion of other environmental variables (Kruuk & Hadfield, 2007). Recently, the development of sequencing techniques and the accompanying increase in genome‐wide association studies (GWAS) offers a third option to estimate heritability, based on the proportion of phenotypic variance explained by all single nucleotide polymorphisms (SNPs), which is known as SNP‐based heritability (Yang et al., 2010). SNP‐based heritability is potentially more accurate, as it is derived directly from the genomic architecture of individuals and is thus less likely affected by confounding environmental effects, or wrongly inferred genealogical relationships. However, SNP‐based heritability could be limited by SNP array size and density, and depending on linkage disequilibrium, SNPs typed might not tag all loci causally linked to the trait, resulting in incomplete estimation of genetic variance (Yang et al., 2017). Despite the increased popularity in GWAS, the use of SNP data in quantitative genetics is still limited, and little comparison has been made between pedigree‐based and SNP‐based heritability, estimates especially in wild populations (but see, e.g., Bérénos et al., 2014).

To date, the sources of heterogeneity among telomere length heritability estimates have not yet been formally examined, and the reliability of existing estimates remains uncertain. Here, we conducted, to the best of our knowledge, the first meta‐analysis of telomere length heritability in vertebrate species. We aimed to calculate the mean telomere length heritability across vertebrate species, as well as to test whether telomere length heritability varies across: (1) phylogeny; (2) species; (3) study; (4) artificial and natural environments; (5) early life and later life; (6) telomere extraction methods; (7) statistical methods; (8) whether individuals had repeated measurements to separate permanent environmental effects; and (9) sex. Furthermore, we also tested for publication bias in telomere length heritability estimates, to assess the validity of any general conclusions drawn from existing studies.

## 2 METHODS

### 2.1 Literature search

We conducted all data collection and analyses using R 4.1.2 (R Core Team, 2021). We performed a systematic literature search for publications up to October 2021 (last publication date = 17th September 2021), using and following the guidelines in *litsearchr* 1.0.0, a package that aims to aid the generation of objective, reproducible and time‐efficient search strategies in systematic reviews, using automated text‐mining and keyword co‐occurrence networks (Grames et al., 2019). First, to obtain a list of highly relevant, ‘golden standard’ articles, we conducted a stringent naïve search on the Web of Science and Scopus, using the Boolean search strings ((TI = ((“telomere” AND [“heritab*” OR “additive genetic varia*”])) NOT TS = (“meta*”)) NOT DT = (Review)) and TITLE(“telomere” AND heritab* OR “additive genetic varia*”) AND NOT TITLE‐ABS‐KEY(“meta*”) AND NOT DOCTYPE(re) respectively. We also extracted a list of 33 relevant references from Dugdale & Richardson, 2018. After combining the two literature lists and removing duplicates with *litsearchr*, we obtained 42 highly relevant articles as the ‘golden standards’.

Next, we extracted possible search terms from these ‘golden standards’, from the authors' keywords, and also from the titles and abstracts. For the latter, we used a Rapid Automatic Keyword Extraction (RAKE) algorithm (Rose et al., 2010). Specifically, we extracted potential terms consisting of at least one word (e.g. ‘heritability’) that occurred at least twice across these articles. Using these potential search terms, we built a keyword co‐occurrence network, where vertices represent the potential search terms, and edges represent an instance where two terms occurred in the same article. We then reduced this network based on node strength, which indicates how well a term is connected to other well‐connected terms, and hence its importance (Grames et al., 2019). We retained terms which cumulatively captured 80% of the total strength of the network, and screened them manually to create the final Boolean search string, which was then passed into Web of Science, with additional modifiers to exclude reviews, conference abstracts, book chapters and retracted articles. The full search string was (((((TS = (((“telomere”) AND (“genet* varianc*” OR “genet* variat*” OR herit* OR inherit*)))) NOT DT = (Review)) NOT AB = (review)) NOT DT = (Meeting Abstract)) NOT DT = (Book Chapter)) NOT DT = (Retracted Publication). This full search returned 822 candidate articles, which included all but one of the ‘golden standards'. We added this missed article (from Dugdale and Richardson (2018)), in addition to two more articles—one study from Bauch et al. (2021) (not yet registered on Web of Science), and one study referenced therein. In total, 825 articles were passed onto the screening process.

### 2.2 Article selection & data extraction

**FIGURE 1:**
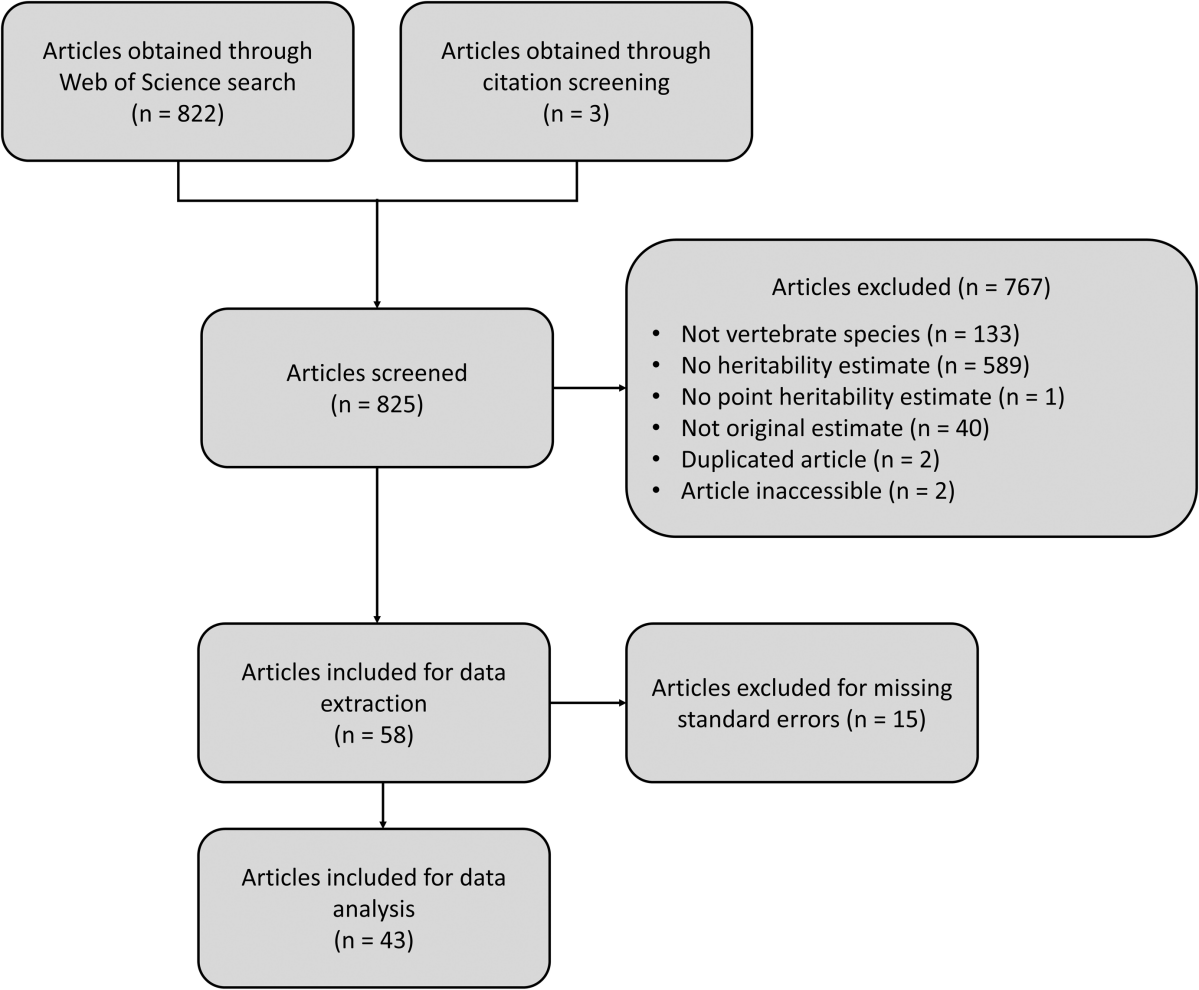
Modified preferred reporting items for systematic reviews and meta‐analyses (PRISMA; Moher et al., 2009) flow chart showing the literature search and screening process

Of these 825 articles, two were inaccessible (Figure 1), and we contacted the authors for the original manuscript. We did not obtain these missing articles and thus excluded them from further selection. We included remaining articles that (1) used a vertebrate species as the subject; (2) reported either the heritability of telomere length, or provided a means to calculate it (e.g. additive genetic variance and phenotypic variance estimates, parent‐offspring regression slopes); (3) reported point heritability estimates as opposed to a range; and (4) reported an original estimate. We employed a double screening process to minimize error, where one author screened through all articles, and a second author independently screened through a random subset of 10% as a control. No discrepancies arose (i.e. Cohen's kappa = 1.0). After screening, 58 articles were retained (Figure 1).

We then either extracted or calculated telomere length heritability estimates from the 58 retained articles. Where an article reported multiple heritability estimates, we extracted all estimates derived from different statistical methods, and the estimate from the fullest model if multiple estimates were derived from the same statistical method. We also included all sex‐specific, time‐specific, subpopulation‐specific or group‐specific estimates to maximize the sample size for statistical analysis. For articles using mid‐parent–offspring regression and/or sex‐specific parent–offspring regression, heritability was taken as the slope, and two times the slope, respectively (Falconer & Mackay, 1996). For articles reporting only 95% confidence intervals for heritability estimates, we calculated the missing standard error using the formula (upper interval limit–estimate)/1.96. We excluded estimates without a standard error or 95% confidence interval. In total, 104 effect sizes from 43 studies and 18 species were used in our analysis (Figure 1).

**FIGURE 2:**
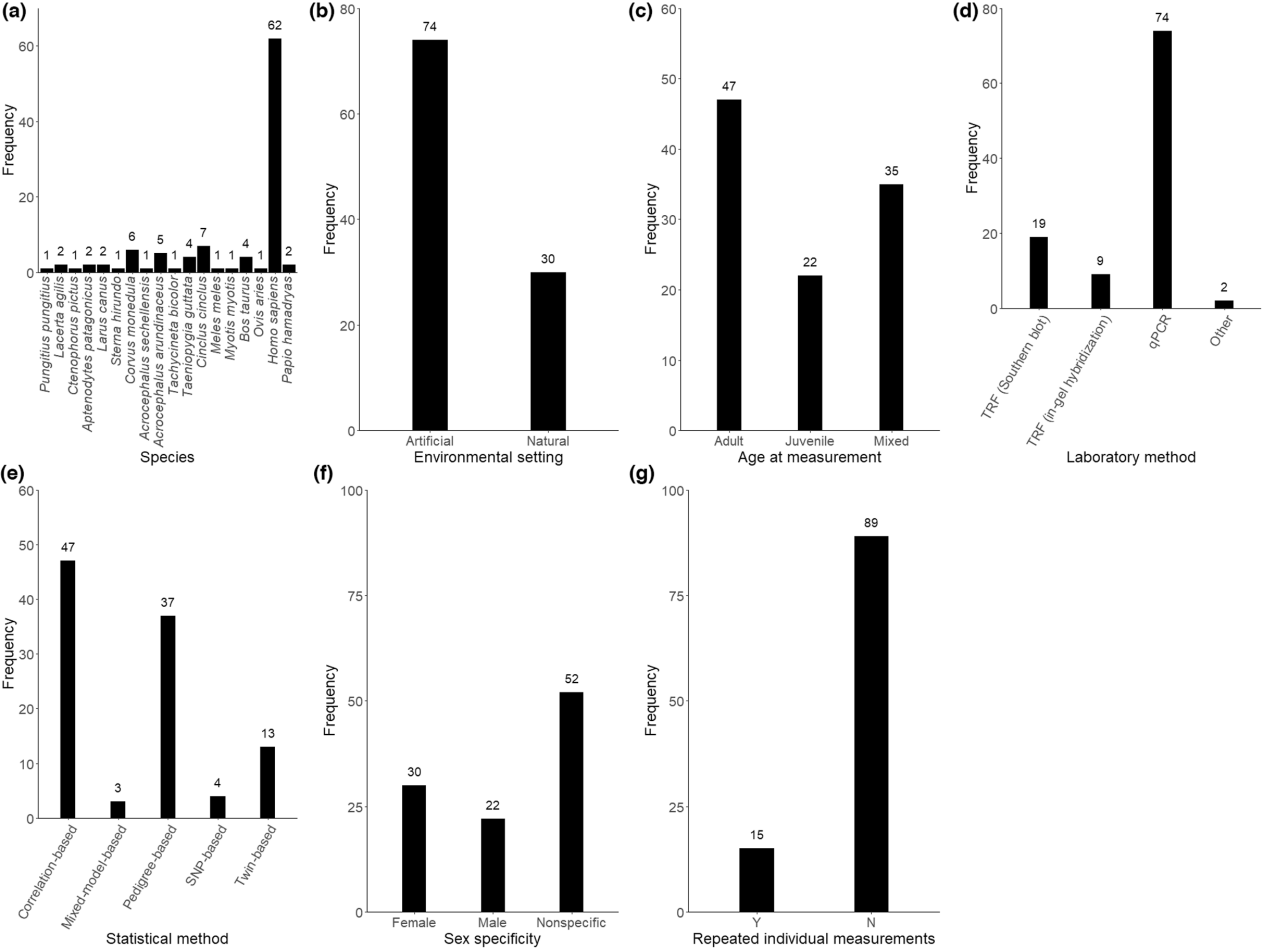
Bar plots showing the distribution of telomere length heritability estimates across (a) species, (b) environmental settings, (c) ages at measurement, (d) laboratory methods, (e) statistical methods, (f) sex specificity and (g) repeated measurements in the study dataset. Numbers above the bars represent sample sizes (*N* = 104).

Each heritability estimate was given a unique estimate ID. Furthermore, for each estimate, we recorded the study ID, species, sample size, environmental setting as a two‐level factor (‘artificial’ for human and captive populations vs ‘natural’ for wild populations), age at measurement as a three‐level factor (‘juvenile’, defined as age before sexual maturity, or ‘adult’ or ‘mixed’), laboratory method as a four‐level factor (‘TRF (Southern blot)’ or ‘TRF (in‐gel hybridization)’ or ‘qPCR’ or ‘other’, with one study using a mix of qPCR and a Luminex‐based assay, and one using a Telomere PNA kit/FITC), and whether there were repeated telomere length measurements for each individual (0/1). We also recorded the statistical methods used to derive heritability and categorized them into five main classes: (1) correlation‐based, that is parent–offspring regression and sibling correlations; (2) pedigree‐based ‘animal models’; (3) twin‐based, that is structural equation modelling or biometric models using monozygotic and dizygotic twin data; (4) SNP‐based, that is genomic‐relatedness‐matrix restricted maximum likelihood models (GREML, Benjamin et al., 2012; Yang et al., 2010), and linkage‐disequilibrium score regression (LDSC, Bulik‐Sullivan et al., 2015); and (5) mixed models, that is models with family ID fitted as a random variable. To account for any potential differences between maternal and paternal inheritance, we recorded parental sex specificity as a three‐level factor (‘Nonspecific’, ‘fathers only’ and ‘mothers only’). The distributions of heritability estimates across these variables are summarized in Figure 2. None of these estimates had any missing data in any moderators. To minimize error, data extraction was conducted by one author, and a second author checked 20% of the extracted estimates. Among the checked data, all heritability estimates were correctly extracted, and there were three discrepancies in the moderators extracted, which were subsequently resolved.

To estimate phylogenetic heterogeneity, we further constructed a phylogenetic tree from the open Tree of Life using *rotl* 3.0.12 (Michonneau et al., 2016), where branch lengths were computed based on Grafen (1989). From this tree, we constructed a phylogenetic correlation matrix using *ape* 5.6.1 (Paradis & Schliep, 2019).

### 2.3 Statistical analysis

We conducted our meta‐analysis using *metafor* 3.0.2 (Viechtbauer, 2010). To estimate the global mean telomere length heritability, we built a meta‐analytic intercept‐only model. We fitted raw heritability estimates as the response variable and used the sampling variance, that is the squared standard error, as the weight for each heritability estimate. Following Nakagawa and Santos (2012), we fitted the following random variables into the model: study ID, to estimate study‐specific effects ($\sigma_{u}^{2})$; the phylogenetic correlation matrix, to test for phylogenetic variation ($\sigma_{a}^{2}$) (Hadfield & Nakagawa, 2010); species, to account for ecological, between‐species variation unrelated to phylogeny ($\sigma_{s}^{2}$); and estimate ID, to test for within‐study, between‐estimate variation ($\sigma_{e}^{2}$). To test for the statistical support of these random effects, we conducted likelihood ratio tests between the full model and one without each of the random variables. We also conducted nonparametric bootstrapping 10 000 times and calculated 95% confidence intervals for all random effects using the percentile method as described in (Davison & Hinkley, 1997) and (Carpenter & Bithell, 2000), with the package *boot 1.3.28* (Canty & Ripley, 2021). From the full model, we also calculated the ‘typical’ sampling‐error variance ($\sigma_{m}^{2}$), that is the within‐study variation from sampling or measurement errors, separated from estimate‐specific effects (Higgins & Thompson, 2002; Nakagawa & Santos, 2012). We calculated the percentage variance explained by each of the random variables, by dividing the respective variance component by the total variance, that is $\sigma_{u}^{2}+\sigma_{a}^{2}+\sigma_{s}^{2}+\sigma_{e}^{2}+\sigma_{m}^{2}$. We then calculated the total heterogeneity (*I* ^2^) estimated from the model by

$$I^{2}=\frac{\sigma_{u}^{2}+\sigma_{a}^{2}+\sigma_{s}^{2}+\sigma_{e}^{2}\;}{\sigma_{u}^{2}+\sigma_{a}^{2}+\sigma_{s}^{2}+\sigma_{e}^{2}+\sigma_{m}^{2}}\;\left(1\right)$$

To investigate the effects of specific biological and methodological factors, we expanded the above model into a meta‐regression and included the following moderators as fixed effects: environmental setting, age at measurement, laboratory method, statistical method and repeated measurements. As heritability estimates could differ between sexes depending on the models used, we added parental sex specificity as an interacting variable with statistical method. This interaction was not significant (Table S1) and was subsequently removed to aid interpretation of first‐order effects.

**FIGURE 3:**
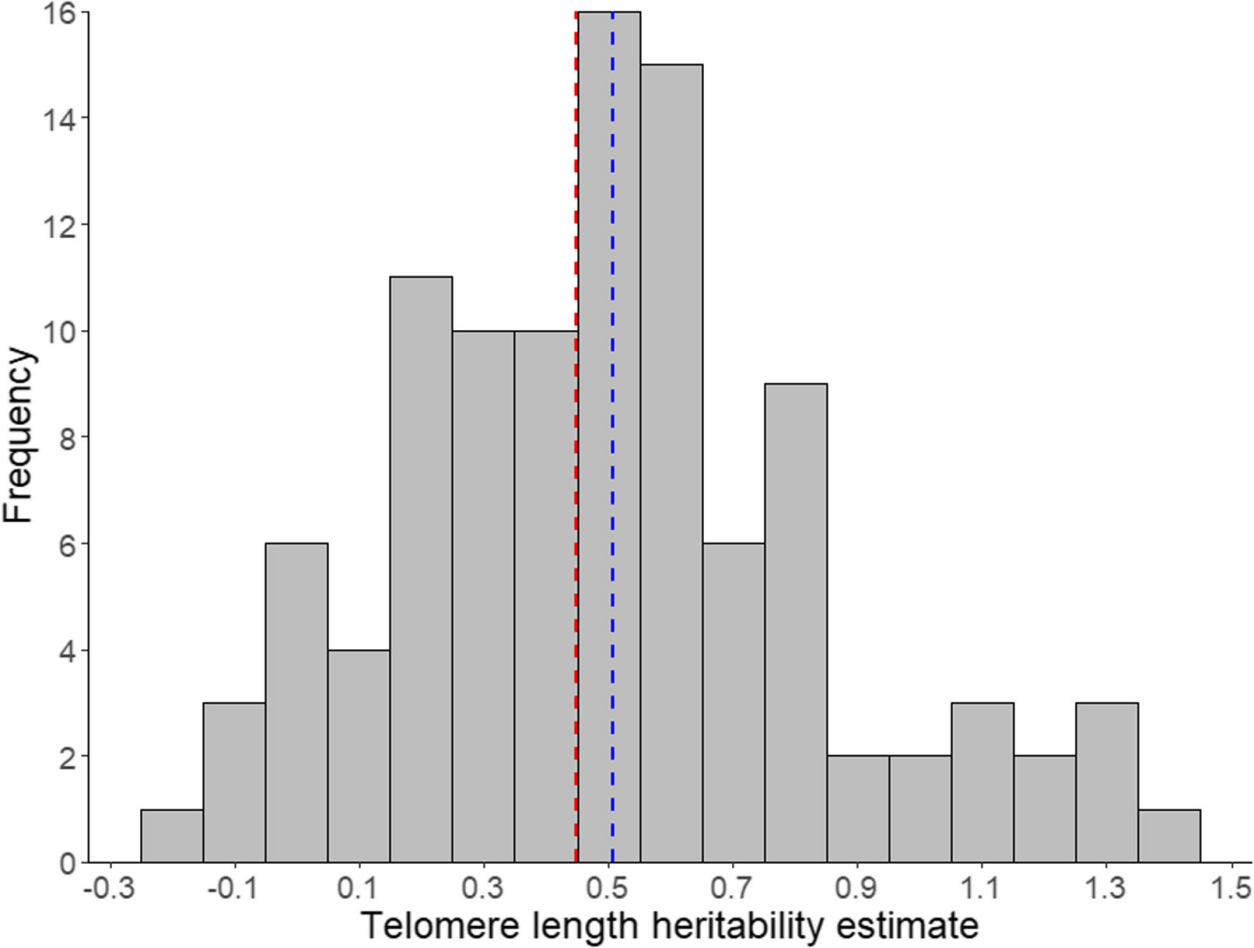
Histogram displaying frequency distribution of telomere length heritability estimates from the dataset (*n* = 104). Blue dashed line indicates unadjusted mean telomere length heritability, red dashed line indicates the mean adjusted for study, phylogenetic, species and estimate effects.

In both models, we fitted raw heritability estimates as the response variable, because the data were close to being normally distributed (Figure 3). Also, as these heritability estimates were derived from various statistical metrics, and spanned more than theory would allow (between 0 to 1 by definition; Figure 3), we decided to not perform any data transformation into common meta‐analytic effect sizes, such as the Fisher's z transformation (e.g. in Dochtermann et al., 2019). We did not exclude estimates outside of the 0 to 1 boundary, as they are useful in examining under‐ or overestimation of heritability, and data trimming could introduce bias in the dataset. Nevertheless, to test for the effects of using untransformed heritability estimates, we conducted the following sensitivity analysis: first, we re‐ran our meta‐regression using Fisher's z‐transformed heritability estimates and their corresponding sampling variances following Dochtermann et al. (2019) (‘Transformed Model’). As estimates greater than one would return undefined values after transformation, we excluded these estimates, as well as those with missing sample sizes and thus sampling variance. In total, 13 estimates were removed. Second, because differences in model results can either be due to data transformation, or the trimming of out‐of‐bound estimates, we ran the meta‐regression again using untransformed estimates from this trimmed dataset (‘Trimmed Model’).

Following Nakagawa et al. (2021), we further examined two types of publication bias: (1) outcome reporting bias, where small studies reporting small or non‐significant effect sizes with low precision might not be published (Nakagawa et al., 2021) and (2) time‐lag bias, where larger effect sizes are published more quickly than smaller or non‐significant effect sizes, creating a decline in effect size magnitudes with time (Koricheva & Kulinskaya, 2019). We visually assessed outcome reporting bias using a funnel plot, which displays effects sizes (in our case telomere length heritability) on the x‐axis, and precision (the inverse of the standard error) on the *y*‐axis. The plot is expected to take the shape of an inverted funnel, and outcome reporting bias would manifest as funnel asymmetry. To construct the funnel plot, we used the residuals from the full meta‐regression model, as using residuals essentially eliminates heterogeneity and effect size non‐independence due to phylogeny, study, etc, which could also produce funnel asymmetry (Nakagawa et al., 2021). To statistically test for this asymmetry, we also ran a variation of Egger's regression (Egger et al., 1997; Fernández‐Castilla et al., 2021; Nakagawa et al., 2021), which allows the incorporation of random effects to account for non‐independence among estimates:

(2) $$y_{i}\sim \beta_{0}+\beta_{1}v_{i}+u_{j}+s_{k}+e_{i}$$

where $y_{i}$ is the *i*th heritability estimate, $v_{i}$ is the corresponding sampling variance following Nakagawa et al. (2021), $u_{j}$ is the between‐study effect of the *j*th study, $s_{k}$ is the between‐species of the *k*th species, and $e_{i}$ is the residual between‐estimate effect. If $\beta_{1}$ is statistically significant, there is evidence of publication bias (Fernández‐Castilla et al., 2021). As previously suggested, the risk of outcome reporting bias could differ between laboratory methods (Wilbourn et al., 2018), and as such, in addition to the whole dataset, we also ran Egger's regressions separately on TRF and qPCR studies. To examine time‐lag bias, we ran a second model by replacing ${\mathrm{se}}_{i}$ with ${\text{year}}_{j}$, the publication year. All publication bias models were run in *metafor*, with sampling variance as weights.

## 3 RESULTS

**FIGURE 4:**
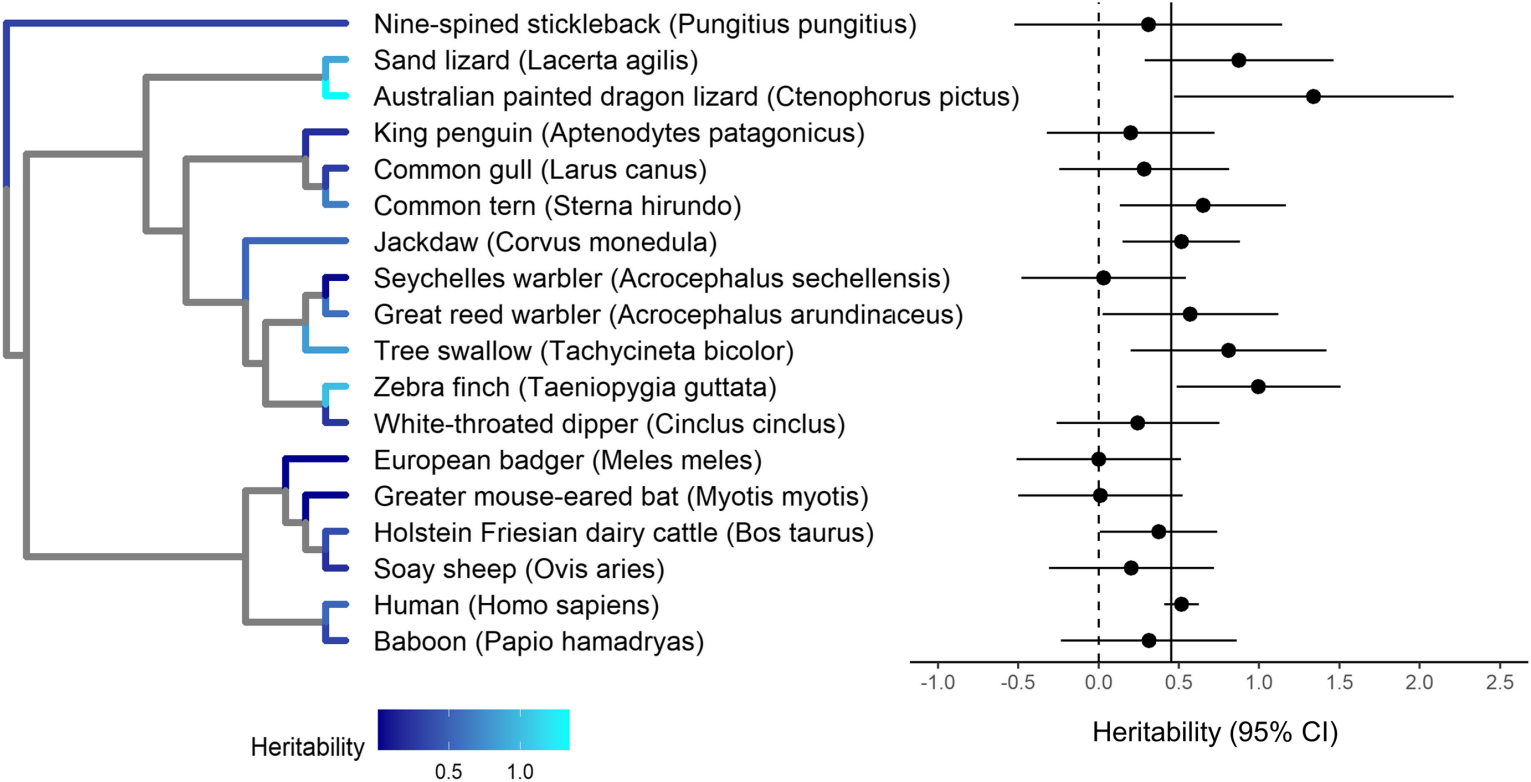
Phylogenetic tree (left) and forest plot (right) of the 18 vertebrate species included in our meta‐analysis, showing the distribution of telomere length heritability estimates across branches. Species‐specific estimates were obtained from a meta‐regression of estimates of each species, accounting for study and estimate effects. In the forest plot, each dot represents the predicted telomere length heritability for each species, and horizontal lines around the dot represent its respective 95% CI. The dotted vertical line on the forest plot indicates zero, whereas the solid vertical line represents the global adjusted mean. In the phylogenetic tree, blue shading indicates predicted telomere length heritability, with a lighter shade representing higher heritability. The phylogenetic tree was visualized using the R package *ggtree* 3.2.1 (Yu et al., 2017).

The mean telomere length heritability in our raw dataset was 50.7% (*SD*: 34.3%, range: −15.6% to 138%). Human (*Homo sapiens*) was the most represented species, accounting for 62 of 96 heritability estimates (60%, Figure 2). After adjusting for phylogenetic, species, study and estimate effects, we found a moderate overall telomere length heritability of 44.9% (95% CI: 25.2–64.7%). The I^2^ calculated from the model was 99.2%, indicating large heterogeneity across heritability estimates. Study ID explained most of this heterogeneity (Table 1), suggesting that telomere length heritability estimates could be influenced by methodological choices in addition to biology. Additionally, we also found phylogenetic and species effects (Table 1, Figure 4), indicating that heritability differences across taxa could also be induced by evolutionary history and ecology. However, likelihood‐ratio tests did not reveal statistically significant support for phylogenetic or species effects, and their 95% confidence intervals bordered zero (Table 1), and hence, our results should be taken with caution.

**TABLE 1:** Variance among studies, phylogeny, species and estimates from the meta‐analytic intercept‐only model of telomere length heritability in 18 vertebrate species, along with ‘typical’ sampling error (i.e. the within‐study variation from sampling or measurement errors, separated from estimate‐specific effects)

Variance component	Estimate	95% CI	No. of levels	Proportion explained	Log‐likelihood	*p*‐Value
**Study ID**	**0.066**	**0.042–0.094**	**43**	**0.650**	**−26.192**	**<0.001**
Phylogeny	0.017	0.000–0.114	18	0.172	−0.679	0.615
Species	0.012	0.000–0.036	18	0.124	−0.706	0.579
**Estimate ID**	**0.005**	**0.000–0.019**	**104**	**0.046**	**−4.345**	**0.006**
‘Typical’ sampling error	0.001		104	0.008		

*Note*: 95% CIs were calculated from nonparametric bootstrapping (*R* = 10 000), using the percentile method. Log‐likelihood and *p*‐values were calculated from likelihood ratio tests by dropping each random effect individually and comparing it to the full model including all effects. Bold text indicates statistically significant results. Total heterogeneity (*I* ^2^) estimated from the model = 0.992.

Looking more closely into the biological and methodological sources of heterogeneity in telomere length heritability, we found that estimates did not differ between environmental settings, age at measurement and whether telomeres were measured multiple times within an individual (Table 2). However, estimates differed between laboratory methods, with TRF (in‐gel hybridization) yielding higher heritabilities than both TRF (Southern blot) and qPCR (Table 2). There were also differences among statistical methods. Compared with correlation‐based methods, twin‐based and SNP‐based models yielded lower heritabilities, which was statistically significant (Table 2). Re‐running the model using SNP‐based methods as the reference level confirmed that SNP‐based heritabilities were lower than both correlation‐ and pedigree‐based estimates, but not twin‐based estimates (difference in intercept with twin‐based methods = 0.249, *SE* = 0.204, *p*‐value = 0.222).

**TABLE 2:** Moderator estimates from a meta‐regression model of telomere length heritability, with non‐significant interaction of parental sex‐specificity removed, and accounting for phylogenetic, species, study and estimate non‐independence

Fixed effects					
	Estimate	*SE*	95% CI	*z*‐Value	*p*‐Value
**(intercept)**	**0.575**	**0.165**	**0.252 to 0.898**	**3.490**	**<0.001**
Environmental setting (Artificial)	−0.181	0.127	−0.431 to 0.069	−1.421	0.155
Age at measurement (Adult)					
Mixed	−0.092	0.093	−0.274 to 0.091	−0.982	0.326
Juvenile	−0.094	0.134	−0.358 to 0.169	−0.702	0.483
Laboratory method (TRF Southern blot)					
**TRF (in‐gel hybridization)**	**0.390**	**0.182**	**0.032 to 0.748**	**2.138**	**0.032**
qPCR	−0.047	0.109	−0.261 to 0.166	−0.433	0.665
Other	0.347	0.270	−0.180 to 0.887	1.292	0.196
Repeated measurement (Yes)	0.146	0.131	−0.110 to 0.403	1.001	0.264
Statistical method (correlation‐based)					
Pedigree‐based	−0.082	0.064	−0.206 to 0.043	−1.283	0.200
Mixed‐model‐based	−0.050	0.242	−0.525 to 0.425	−0.208	0.835
**SNP‐based**	**−0.508**	**0.180**	**−0.862 to −0.154**	**−2.816**	**0.005**
**Twin‐based**	**−0.259**	**0.128**	**−0.509 to −0.008**	**−2.026**	**0.043**

Random effects		
	Estimate	No. of levels
Study ID	0.055	43
Phylogeny	0.000	18
Species	0.000	18
Estimate ID	0.004	104

*Note*: Parentheses in the subheadings of factorial moderators indicate the reference level. Bold text indicates statistically significant results.

From our sensitivity analysis, the Transformed Model using Fisher's *z*‐transformed heritability estimates returned results different from those of our meta‐regression. Specifically, we no longer detected a statistically significant difference between statistical methods, but we detected an environmental setting effect, where estimates from natural studies were lower than those from artificial settings. The laboratory methods effects were retained (Table S2). However, such discrepancies with the untransformed model likely stemmed from data trimming of the 13 out‐of‐bounds heritability estimates, as the Trimmed Model provided qualitatively similar results to the Transformed Model, with no statistical method effect but differences between environmental settings and laboratory methods also observed (Table S3), suggesting that our main model is more sensitive to effect size exclusion than data transformation.

**FIGURE 5:**
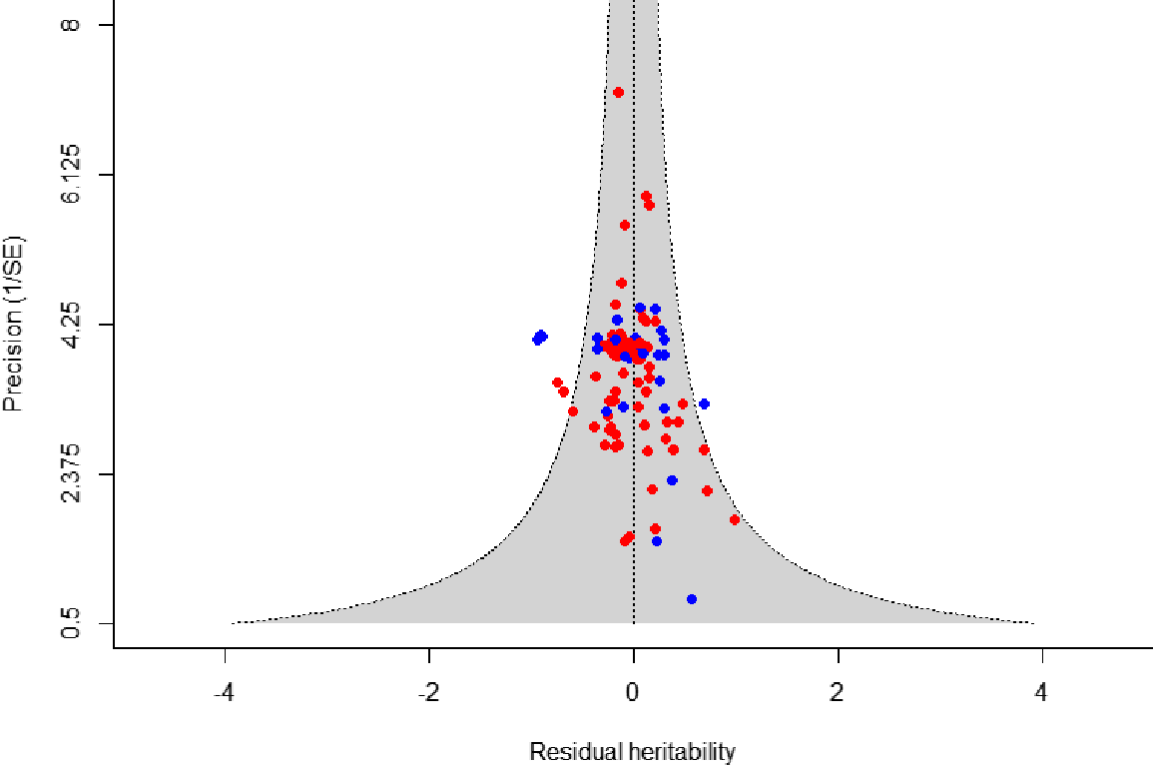
Funnel plot displaying residual telomere length heritability estimates from the full meta‐regression model, against estimate precision. Red dots represent studies using qPCR, and blue dots represent those using TRF methods. The grey area bound by dotted lines represents the 95% confidence interval. Visualized using *metafor* (Viechtbauer, 2010).

Visual examination of the funnel plot suggested truncated effect sizes on the left side, that is a lack of published studies with small sample sizes and/or low precisions reporting small, zero or out‐of‐bound (negative) heritability estimates (Figure 5). However, funnel asymmetry was not detected by weighted Egger's regression using sampling variance as a predictor (*β*_1_ = 0.544, *SE* = 0.498, *p*‐value = 0.274, Table S4), indicating a lack of significant outcome reporting bias. There was also no outcome reporting bias in either qPCR or TRF separate datasets (Figure 5, Tables S5 and S6). Similarly, we did not detect a time‐lag bias using publication year as a predictor in our full dataset ($\beta_{1}=$−0.013, *SE* = 0.010, *p*‐value = 0.184, Table S7).

## 4 DISCUSSION

Telomere length was, on average, moderately heritable (44.9%) in vertebrates. Previously, Karkkainen et al. (2021) reported an individual repeatability of 55% (95% CI: 5–95%) for telomere length in non‐mammal vertebrates. As individual repeatability imposes an upper limit on heritability (Falconer & Mackay, 1996), our heritability estimate was in line with these findings and suggests that in general, the majority of individual telomere length variation is explained by genetic differences rather than permanent environmental variances, as corroborated in other studies (Froy et al., 2021; Sparks et al., 2020). Our global telomere length heritability estimate is higher than that of life‐history traits (26%, Mousseau & Roff, 1987; 33%, Postma, 2014), suggesting that telomere length could be more distant from fitness, and hence is under weaker selection, than other life‐history traits, such as fecundity (Falconer & Mackay, 1996). Although telomere length is correlated with lifespan both phenotypically (Bichet et al., 2020) and genetically (Vedder et al., 2021), it is still unclear how it genetically relates to other fitness components such as reproductive output. Future studies should examine the genetic correlation with these other fitness components, to better quantify the actual selection pressure on telomere length.

We found substantial heterogeneity in telomere length heritability estimates, in particular among studies and estimates. There were potentially phylogenetic and species differences, suggesting effects of shared evolutionary history, such that species more closely related to each other display more similar heritability estimates; or species‐specific ecological effects, such as climate, habitat quality (Angelier et al., 2013; Simide et al., 2016) and/or behaviour (Lewin et al., 2015; Quque et al., 2021). However, our phylogenetic and species results should be interpreted with caution, as both effects were not significant. Only 18 vertebrate species currently have telomere length heritability estimates available, and estimates are heavily skewed. Mammals, in particular humans, and birds were the most researched, whereas other taxa such as non‐avian reptiles, amphibians and fish are severely under‐represented (Figure 2). Therefore, the phylogenetic and species heterogeneity detected could be a result of taxa bias. To reliably determine phylogenetic patterns in telomere length heritability, and thus the evolutionary history of telomere length, further research in these little‐examined taxa is needed.

We also examined the effects of study‐ and estimate‐level factors on telomere length heritability. We found that heritability estimates differed by laboratory methods, and TRF with in‐gel hybridization resulted in higher heritability estimates than both TRF with Southern blotting and qPCR. This again agreed with Karkkainen et al. (2021), who found that telomere extraction technique influenced individual repeatability in telomere length. Compared with TRF, which measures absolute telomere length (Montpetit et al., 2014), qPCR measures telomere length relative to a single‐copy reference gene (Cawthon, 2002; Montpetit et al., 2014) and thus has higher measurement errors and lower inter‐assay consistencies (Aviv et al., 2011; Nettle et al., 2019). In addition, both qPCR and TRF with Southern blotting, which involves the denaturation of DNA, measure also interstitial telomeric sequences (ITS), and the telomeric repeats not located at the end of chromosomes (Foote et al., 2013). On the contrary, TRF with in‐gel hybridization does not involve DNA denaturation and hence excludes ITS in telomere measurement (Atema et al., 2015). As ITS could vary substantially among individuals (Foote et al., 2013), inclusion of ITS measurements can result in inflated total phenotypic variation and thus lower heritability estimates.

More interestingly, we found that heritability estimates significantly differed by statistical method, with correlation‐based methods resulting in the highest mean heritability estimates, and twin‐ and SNP‐based methods being significantly lower. In our dataset, correlation‐based methods include both parent–offspring regressions and sibling correlations. Both methods are based on estimating genetic correlation between pairs of related individuals, and calculating the heritability as the proportion of the observed regression or correlation coefficient to the expected coefficient if the trait is completely inherited (Falconer & Mackay, 1996; Fernandez & Miller, 1985). Although correlation‐based methods are straightforward and less data‐hungry, they are also prone to errors. First, they assume all phenotypic resemblance between genetically correlated individuals is the sole result of inheritance (Falconer & Mackay, 1996). In truth, however, phenotypic resemblance can also result from the shared environment. For example, families living under the same level of pollution experience similar stress‐induced telomere shortening (Bijnens et al., 2015). Unless shared environmental effects are accounted for either statistically (Kruuk & Hadfield, 2007), or by randomizing parent and offspring environment (Fernandez & Miller, 1985), for example by cross‐fostering, they would be confounded with genetic effects, leading to overestimation of heritability. Second, a problem perhaps unique to telomere length parent–offspring regression, is the paradoxical phenomenon that older parents with shorter telomeres were sometimes found to produce offspring with longer telomeres (Bhaumik et al., 2017; Brown et al., 2021; Stindl, 2016), likely due to differential telomere lengths in the germline and somatic cells. This could lead to a negative slope between parent and offspring telomere length, and subsequently negative telomere length heritability, which is, by definition, impossible. One way to correct for this is to account for parental age at conception in the parent–offspring regression model (Dugdale & Richardson, 2018), but this was not always done in our dataset where negative parent–offspring regression slopes were reported.

Alternatively, statistical methods based on variance–covariance partitioning using related individuals could limit these errors. In our dataset, such methods are represented by the ‘animal model’, which estimates heritability by partitioning phenotypic variance into its components, and then estimating and comparing phenotypic covariance between all related pairs in the population using pedigree information (Kruuk & Hadfield, 2007; Lynch & Walsh, 1998; Wilson et al., 2010); as well as structural equation modelling using monozygotic and dizygotic twin data. This method partitions phenotypic covariance within monozygotic and dizygotic twin pairs into additive genetic, dominance, shared environmental and non‐shared environmental components, by exploiting the fact that monozygotic twins share 100% of their genes, whereas dizygotic twins share only 50% (Bischoff et al., 2005; Neale & Maes, 1992). Variance partitioning frameworks allow for environmental effects to be explicitly modelled as random variables, and thus their separation from genetic effects. As such, they are generally expected to have higher accuracy than simple parent–offspring regression (De Araujo & Coulman, 2004; Kruuk & Hadfield, 2007). This could be the reason why we detected significantly lower estimates with twin‐based analysis. Nevertheless, we did not detect a difference between correlation‐based and pedigree‐based estimates as expected. This is likely because the accuracy of pedigree‐based heritability heavily depends on pedigree structure and quality, sample size, and whether environmental effects were explicitly modelled (Bérénos et al., 2014; De Villemereuil et al., 2013; Kruuk & Hadfield, 2007). Links in the pedigree could be incorrectly assigned, for example if parentage is inferred from behaviour instead of from genetic markers, thus failing to correct for extra‐pair paternity, which could be common (Griffith et al., 2002). Furthermore, previous studies showed that the ability of the ‘animal’ model to remove bias was dependent on pedigree depth (Kruuk & Hadfield, 2007). In our dataset, with a heavy skew towards human studies, most pedigree‐based estimates were derived using two to three generations, hence possibly resulting in more biased and inflated estimates. Future work should further assess the effects of pedigree quality to better inform data collection.

On the contrary, SNP‐based heritability estimates were also lower than correlation‐based estimates. In our dataset, SNP‐heritability estimates were calculated using two methods—the GREML method is a linear mixed modelling approach, where the additive effects of all typed SNPs on the phenotype is summarized in a genomic‐relatedness matrix to calculate additive genetic variance (Yang et al., 2010, 2017) and the LDSC method which regresses summary test statistics from typed SNPs against their LD scores, indicating their ability to tag other local variants. The higher the LD score, the higher the probability of a variant tagging causal variants of the trait, and thus, the higher its test statistic would be. The heritability is then derived from the slope of this regression (Bulik‐Sullivan et al., 2015; Yang et al., 2017; Zheng et al., 2017). SNP‐based heritability could potentially be more accurate than that derived from other methods due to several reasons. First, as SNP‐based heritability is calculated directly from genomic data, it encompasses only true additive genetic effects, as opposed to including also dominance and epistasis effects that are rarely separated in other relatedness‐based models, in addition to common environmental effects. This would reduce heritability inflation (Bérénos et al., 2014). Second, when applied to related individuals, SNP‐based heritability is based on realized genetic relatedness, as opposed to expected genetic relatedness in for example pedigree‐based models, and hence is under fewer assumptions, including all non‐genetic effects that could not be modelled (Visscher et al., 2006). Third, SNP‐based heritability is also free from errors due to imprecisely inferred genetic relationships in pedigrees (Faul et al., 2016), or less well‐connected pedigrees. However, as a caveat, SNP‐based methods rely upon the size and the density of the SNP array as well as linkage disequilibrium, as these will determine whether causal loci are tagged by genotyped markers (Manolio et al., 2009; Yang et al., 2017). This would lead to the incomplete capture of variation explained by genetic variants and hence underestimation of heritability. Although some existing studies successfully estimated the heritability of traits using SNP data in a variety of species (e.g. Bérénos et al., 2014; Santure et al., 2013; Visscher et al., 2006), quantitative genetics research using SNP data is still limited, especially in wild animal systems, and more studies will be needed to comprehensively assess and validate its accuracy.

We did not detect any other methodological effects on telomere length heritability estimates. However, it should be noted that the lack of differences between these methodological factors could be due to limited sample sizes and unbalanced data (Figure 2). For example, studies with multiple individual measurements are lacking.

We did not detect any outcome reporting or time‐lag bias from Egger's regression models, despite contrasting conclusions drawn from the funnel plot. This discrepancy is likely a result of the general difficulty in detecting bias from visual methods (Terrin et al., 2005). Furthermore, contrary to the findings by Wilbourn et al. (2018), we did not find a publication bias if we separated TRF and qPCR studies, suggesting that despite differences in accuracy and resource requirements, null results from both methods could be regarded as equally readily publishable by researchers.

In conclusion, we conducted, to our knowledge, the first meta‐analysis on telomere length heritability in vertebrates. We found that telomere length is overall moderately heritable, and there is considerable heterogeneity among heritability estimates, providing grounds for further testing of selection and ecological pressures on telomere length across more diverse study systems. Our findings highlight the importance of the choice of laboratory and statistical method in calculating heritability, and we urge that future studies be mindful of the assumptions and limitations of various methods of estimating heritability. We also recommend that future research should systematically assess the precision and sources of bias of different heritability estimation methods, using both empirical and simulation data, especially in wild, non‐human systems. Furthermore, we noticed an underrepresentation of taxa outside of humans and birds. As literature bias could compromise the validity of meta‐analytical results (Nakagawa et al., 2021; Vevea et al., 2019), we therefore encourage further investigation into more taxa, laboratory and statistical methods, to minimize the possibility of incorrect conclusions that would drive future research.

## AUTHOR CONTRIBUTIONS

HLD, JS and AMS conceived the study. HYJC collected the data and performed the statistical analyses with input from AMS and HLD. HYJC wrote the first draft of the manuscript with input from AMS and JS. All authors contributed to the development of the study, provided comments on the manuscript and agreed on the final version of the manuscript to be submitted for publication.

## CONFLICT OF INTEREST

Please note HYJC has a COI with a deciding editor, JS (co‐author in this manuscript).

## Supporting information


Appendix S1

## Data Availability

Datasets and R code used in this study are openly available in Figshare at https://doi.org/10.6084/m9.figshare.20079026.v1.
