# Supplementary material for: A meta‐analysis on the heritability of vertebrate telomere length
Source: J Evol Biol. 2022 Aug 6;35(10):1283–95. doi: 10.1111/jeb.14071 (PMC9804776; doi:10.1111/jeb.14071)
Supplement: Supplementary file 1 — Appendix S1 [file JEB-35-1283-s001.docx]

Table S1. Moderator estimates from the full meta-regression model of telomere length heritability, accounting for phylogenetic, species, study and estimate non-independence. Since not all statistical methods had sex-specific estimates, not all interactions between statistical method and sex specificity were modelled. Parentheses in the subheadings of factorial moderators indicate the reference level. Bold text indicates statistically significant results.

| **Fixed effects** | | | | | | | |
| --- | --- | --- | --- | --- | --- | --- | --- |
|  |  | | *Estimate* | *s.e.* | *95% CI* | *z-value* | *p-value* |
| **(intercept)** | | | **0.634** | **0.183** | **0.276 – 0.992** | **3.470** | **<0.001** |
| Environmental setting (Artificial) | | | -0.182 | 0.128 | -0.432 – 0.068 | -1.427 | 0.154 |
| Age at measurement (Adult) | | |  |  |  |  |  |
|  | Mixed | | -0.091 | 0.093 | -0.274 – 0.092 | -0.971 | 0.332 |
|  | Juvenile | | -0.098 | 0.133 | -0.359 – 0.164 | -0.734 | 0.463 |
| Laboratory method (TRF Southern blot) | | |  |  |  |  |  |
|  | **TRF (in-gel hybridization)** | | **0.405** | **0.184** | **0.044 – 0.765** | **2.200** | **0.028** |
|  | qPCR | | -0.041 | 0.110 | -0.257 – 0.175 | -0.372 | 0.710 |
|  | Other | | 0.352 | 0.271 | -0.198 – 0.883 | 1.297 | 0.195 |
| Repeated measurement (Yes) | | | 0.132 | 0.132 | -0.126 – 0.390 | 1.001 | 0.317 |
| Statistical method (Correlation-based) | | |  |  |  |  |  |
|  | Pedigree-based | | -0.142 | 0.106 | -0.350 – 0.065 | -1.348 | 0.178 |
|  | Mixed-model-based | | -0.109 | 0.256 | -0.611 – 0.393 | -0.425 | 0.671 |
|  | **SNP-based** | | **-0.558** | **0.199** | **-0.948 – -0.168** | **-2.804** | **0.005** |
|  | **Twin-based** | | **-0.306** | **0.148** | **-0.596 – -0.017** | **-2.074** | **0.038** |
| Sex specificity (Non-specific) | | |  |  |  |  |  |
|  | Mothers only | | -0.039 | 0.091 | -0.217 – 0.139 | -0.431 | 0.667 |
|  | Fathers only | | -0.111 | 0.104 | -0.314 – 0.092 | -1.067 | 0.286 |
| Statistical method: Sex specificity | | |  |  |  |  |  |
|  | Pedigree-based: Mothers only | | 0.053 | 0.109 | -0.161 – 0.267 | 0.486 | 0.627 |
|  | Pedigree-based: Fathers only | | 0.204 | 0.121 | -0.033 – 0.441 | 1.687 | 0.092 |
|  | Twin-based: Mothers only | | 0.055 | 0.134 | -0.207 – 0.317 | 0.413 | 0.680 |
|  |  | |  |  |  |  |  |
| **Random effects** | | | | | |  |  |
|  | | *Estimate* | | | | *No. of levels* | |
| Study ID | | 0.056 | | | | 43 | |
| Phylogeny | | 0.000 | | | | 18 | |
| Species | | 0.000 | | | | 18 | |
| Estimate ID | | 0.004 | | | | 104 | |

Table S2. Moderator estimates from the meta-regression model using z-transformed telomere length heritability estimates, accounting for phylogenetic, species, study and estimate non-independence, with out-of-bounds estimates trimmed out. Models estimates, standard errors, and 95% CIs are presented after back-transformation. Parentheses in the subheadings of factorial moderators indicate the reference level. Bold text indicates statistically significant results. N = 91.

| **Fixed effects** | | | | | | | |
| --- | --- | --- | --- | --- | --- | --- | --- |
|  |  | | *Estimate* | *s.e.* | *95% CI* | *z-value* | *p-value* |
| **(intercept)** | | | **0.675** | **0.254** | **0.302 – 0.869** | **3.162** | **0.002** |
| **Environmental setting (Artificial)** | | | **-0.530** | **0.229** | **-0.782 – -0.132** | **-2.526** | **0.012** |
| Age at measurement (Adult) | | |  |  |  |  |  |
|  | Mixed | | 0.007 | 0.142 | -0.267 – 0.280 | 0.048 | 0.961 |
|  | Juvenile | | 0.238 | 0.279 | -0.310 – 0.667 | 0.845 | 0.398 |
| Laboratory method (TRF Southern blot) | | |  |  |  |  |  |
|  | **TRF (in-gel hybridization)** | | **0.553** | **0.264** | **0.090 – 0.819** | **2.294** | **0.022** |
|  | qPCR | | -0.248 | 0.157 | -0.511 – 0.058 | -1.596 | 0.110 |
|  | Other | | 0.025 | 0.453 | -0.732 – 0.755 | 0.052 | 0.959 |
| Repeated measurement (Yes) | | | 0.029 | 0.192 | -0.338 – 0.388 | 0.150 | 0.880 |
| Statistical method (Correlation-based) | | |  |  |  |  |  |
|  | Pedigree-based | | 0.132 | 0.107 | -0.076 – 0.330 | 1.242 | 0.214 |
|  | Mixed-model-based | | -0.092 | 0.304 | -0.609 – 0.480 | -0.293 | 0.769 |
|  | SNP-based | | -0.349 | 0.277 | -0.727 – 0.192 | -1.279 | 0.201 |
|  | Twin-based | | -0.263 | 0.182 | -0.558 – 0.092 | -1.462 | 0.144 |
|  |  | |  |  |  |  |  |
| **Random effects** | | | | | |  |  |
|  | | *Estimate* | | | | *No. of levels* | |
| Study ID | | 0.096 | | | | 39 | |
| Phylogeny | | 0.012 | | | | 17 | |
| Species | | 0.000 | | | | 17 | |
| Estimate ID | | 0.049 | | | | 91 | |

Table S3. Moderator estimates from a meta-regression model of raw telomere length heritability, accounting for phylogenetic, species, and study non-independence. This model is identical to that in Table 2 with the out-of-bounds estimates trimmed out to test for trimming effects on the results of the z-transformed data (Table S2). Parentheses in the subheadings of factorial moderators indicate the reference level. Bold text indicates statistically significant results. N = 91.

| **Fixed effects** | | | | | | | |
| --- | --- | --- | --- | --- | --- | --- | --- |
|  |  | | *Estimate* | *s.e.* | *95% CI* | *z-value* | *p-value* |
| **(intercept)** | | | **0.634** | **0.154** | **0.332 – 0.937** | **4.116** | **<0.001** |
| **Environmental setting (Artificial)** | | | **-0.325** | **0.145** | **-0.609 – -0.041** | **-2.244** | **0.025** |
| Age at measurement (Adult) | | |  |  |  |  |  |
|  | Mixed | | -0.095 | 0.091 | -0.278 – 0.088 | -1.018 | 0.308 |
|  | Juvenile | | 0.124 | 0.190 | -0.248 – 0.496 | 0.653 | 0.514 |
| Laboratory method (TRF Southern blot) | | |  |  |  |  |  |
|  | **TRF (in-gel hybridization)** | | **0.385** | **0.168** | **0.056 – 0.715** | **2.290** | **0.022** |
|  | qPCR | | -0.082 | 0.102 | -0.282 – 0.118 | -0.804 | 0.421 |
|  | Other | | 0.133 | 0.296 | -0.447 – 0.713 | 0.449 | 0.653 |
| Repeated measurement (Yes) | | | -0.012 | 0.131 | -0.269 – 0.244 | -0.093 | 0.926 |
| Statistical method (Correlation-based) | | |  |  |  |  |  |
|  | Pedigree-based | | 0.002 | 0.067 | -0.129 – 0.134 | 0.036 | 0.972 |
|  | Mixed-model-based | | -0.083 | 0.234 | -0.541 – 0.375 | -0.357 | 0.721 |
|  | SNP-based | | -0.313 | 0.178 | -0.663 – 0.134 | -1.758 | 0.079 |
|  | Twin-based | | -0.145 | 0.119 | -0.378 – 0.036 | -1.214 | 0.225 |
|  |  | |  |  |  |  |  |
| **Random effects** | | | | | |  |  |
|  | | *Estimate* | | | | *No. of levels* | |
| Study ID | | 0.042 | | | | 39 | |
| Phylogeny | | 0.000 | | | | 17 | |
| Species | | 0.000 | | | | 17 | |
| Estimate ID | | 0.004 | | | | 91 | |

Table S4. Summary of the weighted Egger’s regression model for the detection of outcome reporting bias on telomere length heritability, using standard error as a fixed effect, accounting for study and species non-independence. Bold text indicates statistically significant results.

| **Fixed effects** | | | | | | | |
| --- | --- | --- | --- | --- | --- | --- | --- |
|  |  | | *Estimate* | *s.e.* | *95% CI* | *z-value* | *p-value* |
| **(intercept)** | | | **0.436** | **0.060** | **0.318 – 0.554** | **7.262** | **<0.001** |
| Sampling variance | | | 0.544 | 0.498 | -0.431 – 1.520 | 1.094 | 0.274 |
|  |  | |  |  |  |  |  |
| **Random effects** | | | | | |  |  |
|  | | *Estimate* | | | | *No. of levels* | |
| Study ID | | 0.072 | | | | 43 | |
| Species | | 0.009 | | | | 18 | |

Table S5. Summary of the weighted Egger’s regression model, within studies using TRF to measure telomere length, for the detection of outcome reporting bias on telomere length heritability, using standard error as a fixed effect, accounting for study and species non-independence. Bold text indicates statistically significant results. N = 28.

| **Fixed effects** | | | | | | | |
| --- | --- | --- | --- | --- | --- | --- | --- |
|  |  | | *Estimate* | *s.e.* | *95% CI* | *z-value* | *p-value* |
| **(intercept)** | | | **0.606** | **0.100** | **0.410 – 0.802** | **6.061** | **<0.001** |
| Sampling variance | | | 0.152 | 0.697 | -1.244 – 1.519 | 0.219 | 0.827 |
|  |  | |  |  |  |  |  |
| **Random effects** | | | | | |  |  |
|  | | *Estimate* | | | | *No. of levels* | |
| Study ID | | 0.082 | | | | 13 | |
| Species | | 0.011 | | | | 6 | |

Table S6. Summary of the weighted Egger’s regression model, within studies using qPCR to measure telomere length, for the detection of outcome reporting bias on telomere length heritability, using standard error as a fixed effect, accounting for study and species non-independence. Bold text indicates statistically significant results. N = 74.

| **Fixed effects** | | | | | | | |
| --- | --- | --- | --- | --- | --- | --- | --- |
|  |  | | *Estimate* | *s.e.* | *95% CI* | *z-value* | *p-value* |
| **(intercept)** | | | **0.274** | **0.070** | **0.137 – 0.413** | **3.903** | **<0.001** |
| Sampling variance | | | 0.836 | 0.713 | -0.560 – 2.234 | 1.174 | 0.240 |
|  |  | |  |  |  |  |  |
| **Random effects** | | | | | |  |  |
|  | | *Estimate* | | | | *No. of levels* | |
| Study ID | | 0.040 | | | | 28 | |
| Species | | 0.022 | | | | 12 | |

Table S7. Summary of the weighted Egger’s regression model for the detection of time-lag bias on telomere length heritability, using publication year as a fixed effect, accounting for study and species non-independence. Bold text indicates statistically significant results.

| **Fixed effects** | | | | | | | |
| --- | --- | --- | --- | --- | --- | --- | --- |
|  |  | *Estimate* | | *s.e.* | *95% CI* | *z-value* | *p-value* |
| **(intercept)** | | 27.139 | | 20.078 | -12.213 – 66.491 | 1.352 | 0.176 |
| Year | | -0.013 | | 0.010 | -0.033 – 0.006 | 1.328 | 0.184 |
|  |  |  | |  |  |  |  |
| **Random effects** | | | | | |  |  |
|  | | | *Estimate* | | | *No. of levels* | |
| Study ID | | | 0.078 | | | 43 | |
| Species | | | 0.000 | | | 18 | |
